# Human–Wildlife Conflict: The Human Dimension of European Bison Conservation in the Bieszczady Mountains (Poland)

**DOI:** 10.3390/ani11020503

**Published:** 2021-02-15

**Authors:** Daniel Klich, Rafał Łopucki, Magdalena Perlińska-Teresiak, Agata Lenkiewicz-Bardzińska, Wanda Olech

**Affiliations:** 1 Department of Animal Genetics and Conservation, Warsaw University of Life Sciences-SGGW, Ciszewskiego 8, 02-786 Warsaw, Poland; magdalena_perlinska_teresiak@sggw.edu.pl (M.P.-T.); wanda_olech_piasecka@sggw.edu.pl (W.O.); 2 Centre for Interdisciplinary Research, The John Paul II Catholic University of Lublin, Konstantynów 1J, 20-708 Lublin, Poland; lopucki@kul.lublin.pl; 3 Cisna Forest District, Cisna 87A, 38-607 Cisna, Poland; agata.lenkiewicz-bardzinska@krosno.lasy.gov.pl

**Keywords:** European bison, attitude, Carpathians, human–wildlife conflict, damage, forest, village, city, compensation, health risk

## Abstract

**Simple Summary:**

The study aimed to compare the attitudes to European bison of local village inhabitants in Bieszczady and city dwellers in Rzeszów. Our study showed that not only does the growing European bison population cause an increase in negative attitudes among local village communities, but this species also causes more conflict than any other herbivore in the Bieszczady Mountains. Village residents believed that the main threats that arise from the European bison were from the damage they cause and forest use limitations. The current compensation system for the damage caused by this species does not solve this problem, because over 60% of damage is not effectively reported to the state administration. The city dwellers of Rzeszów displayed a different attitude towards the European bison. We concluded that while educational workshops for local villagers may alleviate conflict in the short term, ultimately it is only by restricting the growth of the European bison population that a long-term effect will be achieved.

**Abstract:**

An important limitation for the population growth of European bison in the Bieszczady Mountains may be the level of social acceptance. The study aimed to compare attitudes to European bison of local village inhabitants in Bieszczady and city dwellers in Rzeszów. We also investigated whether damage caused by European bison or other wild species changes peoples’ perceptions of this animal. Our study showed that not only does the growing European bison population cause an increase in negative attitudes among local village communities, but this species also causes more conflict than any other herbivore in the Bieszczady Mountains. Village residents believed that the main threats that arise from European bison were the damage they cause and forest use limitations. The current compensation system for the damage caused by this species does not solve the problem because over 60% of damage is not effectively reported to the state administration. The city dwellers of Rzeszów displayed a different attitude towards the European bison. We concluded that while educational workshops for local villagers may alleviate conflict in the short-term, ultimately it is only by restricting the growth of the European bison population that a long-term effect will be achieved.

## 1. Introduction

The European bison (*Bison bonasus)* has been successfully restored since its total extinction in the wild [1,2], and the most distinct changes have been observed over approximately the last decade [2,3]. Nevertheless, the conservation of this species remains a real challenge for conservationists [4].

The primary problem is the European bison’s low genetic variability, which causes low resistance to infectious and parasitic diseases [5,6,7,8,9]. Moreover, many of the European bison’s health problems are related to their population density. Free-living populations are rare but usually quite large [3]. These populations are not usually spatially connected with each other [10], and the spontaneous colonization of new suitable areas is rare [11], which results in the carrying capacity of forest complexes being exceeded [12,13]. This has led to some populations using habitats outside of forest complexes [14], thus resulting in the following health and economic consequences: a threat to the European bison’s health from pesticides used to protect crops [15]; physiological changes in the mineral status of animals that feed on crops [16]; economic losses experienced by farmers; and less social acceptance of this species by local communities [17,18]. 

The problem of local communities’ attitudes to conservation measures is important because they can impact the success of reintroducing species [19]. For this reason, along with other activities aimed at the conservation of the European bison [20], surveys of local communities have been carried out in order to identify changes in perceptions of the European bison [18,21]. However, there remains little detailed research on the social acceptance of existing European bison populations by local communities. Some studies show that acceptance is high, but this is site-specific and is affected by the close proximity of European bison to respondents [22,23]. However, close living proximity does not explain the differences in perception of the European bison between city dwellers and village inhabitants, whereas such differences have been described for other mammal species [24,25]. An important factor seems to be European bison population management methods and damage to agricultural crops. A study in northeastern Poland showed that in Borecka forest, where crop depredation by European bison was rarely noticed and animals were supplied with food during winter, local communities presented a higher acceptance level to the European bison compared to communities from Knyszyńska forest, where winter feeding was ineffective and frequent crop depredation was observed [18]. However, the issue of the direct impact of the damage caused by European bison on human attitudes has not been directly studied.

There is one population of European bison in the Polish mountains: it is in the Bieszczady Mountains in the northern part of the Eastern Carpathians, near the border with the Slovak Republic and Ukraine. This European bison population in Bieszczady is unique to Polish conditions because it is the only the lowland–Caucasian genetic line that is living in a mountainous landscape with specific local conditions, and it is geographically distant from other free-living European bison populations [16,26,27]. This population is the basis of the concept of creating a European bison metapopulation in the Carpathians that is spatially connected to the populations of Ukraine, Slovakia and Romania [28].

The population in the Bieszczady Mountains was established the 1960s in depopulated areas after World War II [27,29]. After more than 50 years of population growth, during which the population was supplied by relocated animals from other countries [2,30], their numbers have increased (over the last ten years) much more than those of the neighboring Carpathian populations [3,31]. Currently, the Bieszczady Mountains is home to over 660 animals; this not only the second largest European bison population in the world (after the Białowieża Forest population), but it is also the largest population of the lowland–Caucasian line [3]. Given the past and present threats of infectious diseases, this high density is risky [32,33]. Social acceptance may also be an important limitation for further population development. So far, local inhabitants’ attitudes to this population growth are not well known, but recent years have shown increasing damage from European bison in the Bieszczady Mountains [34]; however, according to the official data of the Regional Directorate for Environmental Protection, this damage has not been of great economic value. According to Paszkiewicz and Karaś [35], local inhabitants spoke about the nuisance this species causes, but no studies have attempted to better understand the specific reasons for this perception of the European bison. 

This study presents a survey conducted among inhabitants of areas with a European bison population in the Bieszczady Mountains, as well as among inhabitants of Rzeszów (the capital of this region). We aimed to compare attitudes to the European bison (the importance of their presence for the local community and assessment of population size) between local inhabitants in Bieszczady and city dwellers. We also aimed to compare the views of inhabitants in the Bieszczady Mountains against those of Rzeszów inhabitants concerning the threats and limitations caused by the European bison population in Bieszczady. Another aim was to determine if the damage caused by European bison or other wild mammals impacted people’s perception of this species. We hypothesized that different social groups would have different attitudes and would recognize different threats caused by this species. Moreover, we hypothesized that the damage caused by European bison will directly influence local communities’ perceptions. Based on the literature data [18,23,36], we assumed that revealing and documenting these differences is the basic challenge facing the creation of management strategies under specific situations of the potential human–bison conflict.

## 2. Materials and Methods 

**Figure 1:**
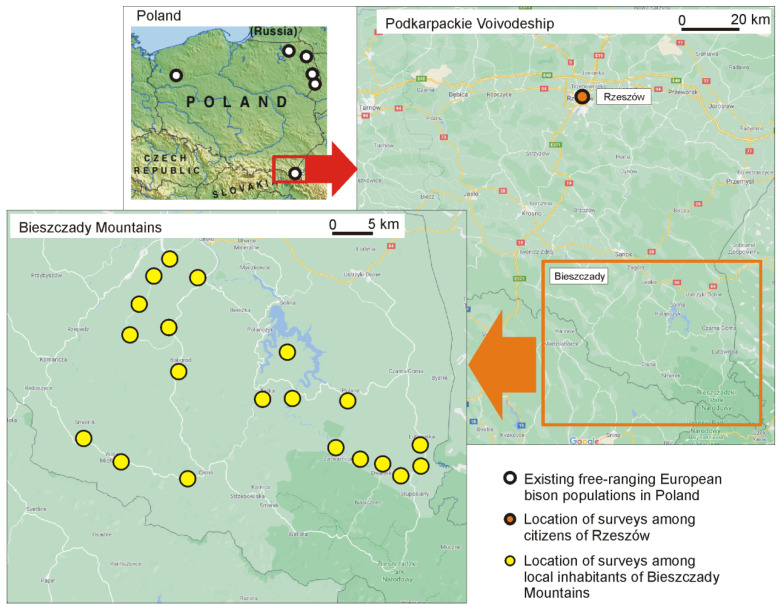
Location of sites where surveys were conducted: the city of Rzeszów (the capital of Podkarpackie Voivodeship) and 20 villages in the Bieszczady Mountains.

The study was performed in southeastern Poland in two locations: (1) the Bieszczady Mountains, i.e., 20 villages in the vicinity of the Baligród Forest District and the Lutowiska Forest District; and (2) the city of Rzeszów, i.e., inhabitants of the capital of Podkarpackie Voivodeship (Figure 1). A total of 401 questionnaires were conducted, of which 301 were in Bieszczady and 100 were in Rzeszów. Respondents were asked direct questions via direct interviews that are usually adopted for qualitative data collection [37]. Interviews were held among randomly chosen inhabitants of Bieszczady in their own homes. Interviews among randomly selected Rzeszów city dwellers were conducted in the city center at random times. The respondents’ age and sex were the only selection criteria; only adult people aged 18 and over were surveyed. We ensured that each age group and each gender group was represented by at least 20% and 40% of respondents, respectively. All respondents were informed about the purpose of the survey and consented to its conduct. The surveys were not linked to respondents’ addresses and were anonymous, therefore no sensitive data was collected.

For the purpose of the study, 14 questions were asked, most of which were closed-ended. Two questions formed the basis of this analysis: (1) “Is the presence of the European bison in the Bieszczady Mountains beneficial for the local community?”; (2) “How many European bison should be in the Bieszczady Mountains? (questions 1 and 2, Table S1).

In both locations, three questions were also asked to clarify the possible disadvantages of the European bison population: these concerned health risks, economic risks, and forest use limitations (questions 3, 4 and 5, Table S1). A choice of five answers was offered for each of the above questions (from “definitely not” or “definitely less” to “definitely yes” or “definitely more”). In order to assess how the damage caused by the European bison and other wildlife influenced their attitudes towards this species, the inhabitants of the villages in the Bieszczady Mountains were asked some additional questions about these issues. Additional questions were also asked about whether single instances of damage had been reported and, if so, what the outcome of this process was. A detailed set of questions is presented in Supplementary Table S1.

The statistical analysis was performed after converting the answers to a numerical 1–5 Likert scale or grouping the variables (Table S1). A comparison of the differences in attitudes to the European bison between the local inhabitants of Bieszczady and the city dwellers of Rzeszów was performed with the U Mann–Whitney test. Comparisons were made between the answers to the two basic questions concerning the presence of the European bison and their population numbers (questions 1 and 2).

We conducted a comparison of the perceived disadvantages or threats caused by the European bison population. This was carried out by using a generalized linear model in which the dependent variable was the answer to question 1 (“Is the presence of the European bison in the Bieszczady Mountains beneficial for the local community?”) and the explanatory variables were health risk, economic risk and forest use limitations (answers to questions 3, 4 and 5). Answers from respondents in both locations were analyzed in the same way. 

Answers from the respondents in the Bieszczady Mountains relating to the number of animals in this region (i.e., question 2) were analyzed whilst taking into consideration local factors such as (A) damage by European bison to the respondent’s property, (B) damage by European bison to the property of the respondent’s family or friends, (C) damage by other wildlife to the respondent’s property, (D) age, (E) sex, and (F) whether the respondent is a hunter. The three variables referencing damage (A, B and C) were transformed into two groups of answers: “YES, damage occurred”, and “NO, there was no damage, or I do not remember”. Age was based on the following groups: 18–39, 40–60, and over 60 years. Hunters were treated as a separate group from other respondents. 

The model selection procedure was similar for all models. We used normal distribution, and the identity link function was based on the Akaike information criterion (AIC) value and the model’s assumptions. Model selection was based on AIC values in a multi-model selection procedure [38]. All possible model permutations with all the explanatory variables (and the null model) were performed; finally, the models were ranked according to their Akaike weights. The principle of model selection was lower AIC values, but when the difference between the models was less than AIC = 2, the simpler model was chosen. A pairwise comparison with Bonferroni adjustment of groups was used for factors that were statistically significant in the model. All statistical analyses were performed using SPSS software (version 24.0, IBM Corporation, Armonk, NY, USA).

## 3. Results

**Figure 2:**
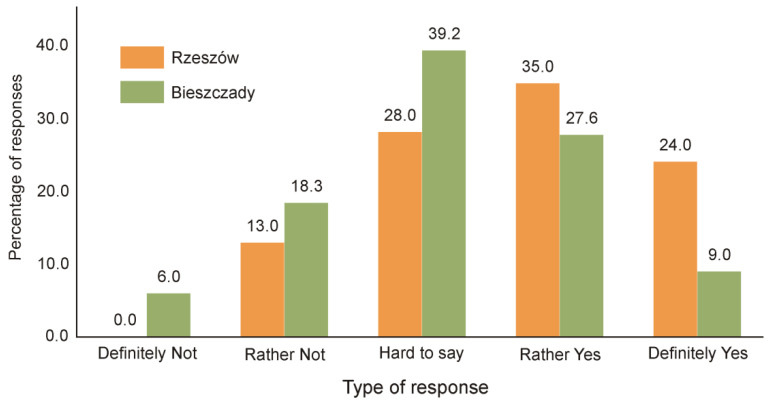
Answers to question 1: “Is the presence of European bison in the Bieszczady Mountains beneficial for the local community?”.

The inhabitants of Bieszczady showed less approval for the European bison population compared to the Rzeszów city dwellers. Almost a quarter of respondents had the opinion that the presence of bison is not beneficial for local communities (“definitely not” or “rather not”), while only 13% of Rzeszów inhabitants expressed such an opinion (Figure 2). At the same time, 37% of the village inhabitants of Bieszczady and almost 60% of the city inhabitants of Rzeszów expressed a positive opinion concerning the presence of European bison. The groups of respondents differed statistically in their attitudes to the presence of European bison (Z = −2.35, *p* < 0.001).

**Figure 3:**
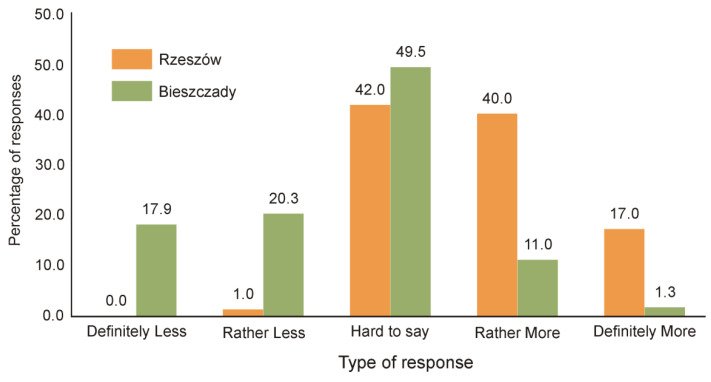
Answers to question 2: “How many European bison should be in the Bieszczady Mountains?”.

A greater difference between city dwellers and local village inhabitants was demonstrated in relation to views on the size of the European bison population in the Bieszczady Mountains. Almost 40% of the Bieszczady village inhabitants stated that there are too many wisents (i.e., there should be “definitely less” or “rather less” individuals), while only 1% of Rzeszów inhabitants held this opinion (Figure 3). Moreover, over half of the city dwellers (57%) stated that there should be more or definitely more European bison in the Bieszczady Mountains, while only 12% of local Bieszczady inhabitants agreed with this statement (Figure 3). The groups of respondents differed statistically in terms of their attitudes to European bison numbers (Z = −9.87, *p* < 0.001).

Assessments of the impact of individual threats related to the European bison population in the Bieszczady Mountains were also different (Table 1). Among the inhabitants of the Bieszczady villages, economic risks and forest use limitations had a significant impact on opinions concerning whether the presence of the bison is beneficial. Forest use limitations seem to be a more important factor that explains opinions on the impact of the European bison population on local communities (a higher value of B coefficient). The city dwellers were not aware of these threats, and only their health-related concerns significantly explained their attitudes to the European bison. All trends were negative, i.e., the higher the assessment of a given risk, the worse the community believed the bison impacted their community.

**Figure 4:**
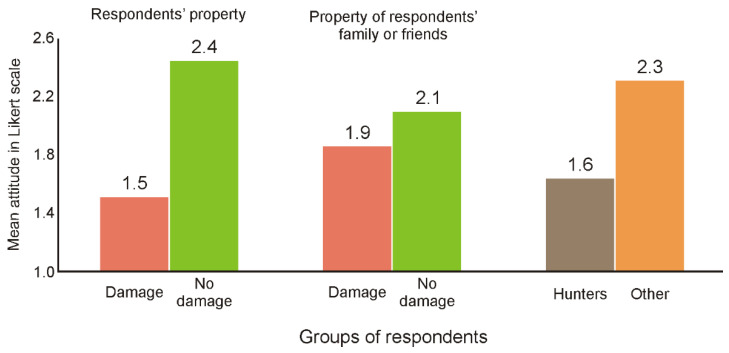
The mean value of attitudes to the European bison population numbers with regard to the following factors: damage to respondents’ property; damage to property of respondents’ family or friends; being a hunter; and pairwise comparison with Bonferroni adjustment.

Analysis of the Bieszczady inhabitants’ attitudes to European bison population numbers showed that damage caused by wisents to the property of respondents (Χ^2^ = 38.01, *p* < 0.001) or their family or friends (Χ^2^ = 5.12, *p* = 0.024) significantly affected attitudes to this species. Neither damage caused by other wild animal species nor the sex or age group of respondents were significant and were therefore excluded during the model selection. However, the attitudes of hunters to European bison numbers were very different than those of non-hunters (Χ^2^ = 15.63, *p* < 0.001); in comparison to other respondents, they indicated significantly more frequently that European bison numbers are too high (Figure 4).

**Figure 5:**
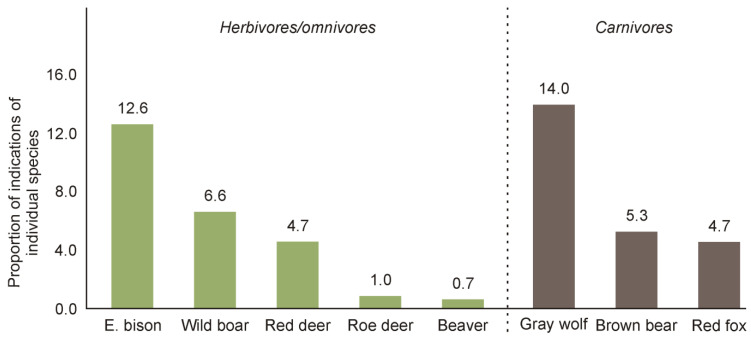
The frequency of indications that individual wildlife species had caused damage to respondents’ property in the previous two years. Species are separated according to primary food source. Each bar represents the percentage of respondents who indicated a given species as causing damage.

Compared to damage caused by other animals, damage caused by European bison was more frequently mentioned by respondents. Over 12% of respondents indicated that the European bison had caused damage to their farms; this is the highest value among all herbivores in this area. Only the wolf, which is considered to cause the greatest nuisance to life in the Bieszczady Mountains, presented a comparable value (Figure 5).

**Figure 6:**
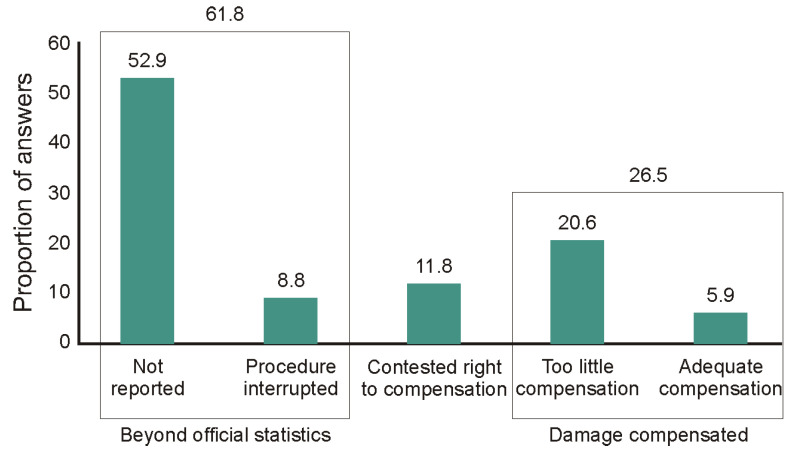
The result of all identified damage by European bison. Most incidents of damage (61.8%) are not reported effectively and therefore are not included in official statistics. Only slightly more than a quarter of damages (26.5%) received compensation from the state administration.

Results from individual incidents of damage by European bison show that most were not reported effectively (62% of cases were not reported, or the complaint procedure was unfinished); they therefore remained outside of official statistics. Some compensation applications (11.8%) were rejected by the state administration, and ultimately only slightly more than a quarter of claims (26.5%) received compensation from the state. A small percentage of respondents (6%) were satisfied with the amount of compensation (Figure 6).

## 4. Discussion

Currently, increasing attention is paid to the impact of people’s views on wildlife management as well as the fact that the survival of many endangered species depends on the willingness of communities to coexist with them [36,39,40,41,42,43]. In this paper we showed that this problem is also very important for the conservation of free-living populations of European bison in the mountainous landscape of Bieszczady. In this study area, the positive effects of many years of conservation activities and the growth of the European bison population are clearly visible [3]. As suggested in previous studies, however, the growing population of this large herbivore is causing an increase in the number of conflict situations, thus threatening a decrease in local communities’ willingness to coexist with them [34,35,36]. We have shown that human dimensions in the management of European bison in Bieszczady are multifaceted and require greater effective integration of natural and social sciences. 

The social perception of the European bison is the first basic issue; there is a discrepancy between the attitudes of local communities (i.e., people who may suffer real consequences of the European bison’s presence in their daily lives) and the attitudes of city dwellers (i.e., those who mainly obtain information about wildlife from mass media). The differences between these social groups in their perceptions of wild animals, including ungulates, are often described in the literature [24,25,36,43,44,45,46]. Therefore, the overall result of our study is not surprising. As expected, city residents had a more positive opinion concerning the benefits of the presence of the European bison in the Bieszczady Mountains (59% positive answers) compared to local village residents (29%). The city residents also more frequently agreed that there should be more European bison in this region) compared to local village residents (57% vs. 12%, respectively). Such highly positive opinions among city dwellers concerning European bison in the Bieszczady Mountains is the result of a generally positive media attitude towards European bison in Poland. In this country, the European bison is considered a symbolic and charismatic species, and it has a high status in culture [26,47].

Studies on the social issues of nature conservation often point out that city dwellers more easily and uncritically declare the need to conserve species or ecosystems because the costs and consequences of this conservation usually do not directly concern them [24,36,48]. People are likely to support the conservation of the European bison, but they prefer not to experience the presence of this animal in the vicinity of their homes [23]. Some authors tried to solve this problem by asking city respondents if they would be willing to contribute towards the cost of the nature projects they support [49], but our survey did not include this option. So, it would appear that the surveyed Rzeszów inhabitants’ opinions on European bison were based mainly on common knowledge about this species; these results are similar to those of Herman et al.’s [50] studies on the attitudes of students towards European bison and wolves in Germany. Evidence for this is the fact that city respondents felt that the greatest threat posed by the bison was to public health (Table 1), which has nothing to do with the actual data on the damage and threats caused by wisents in the Bieszczady Mountains [33]. The actual and most common problems for local residents (such as economic losses or forest use restrictions) are little known to urban respondents (they very rarely indicated them as significant in the survey). Similar phenomena, namely opinions being based on myths and unscientific media messages (including even cartoons), have also been described in other studies about various species conflicts in other countries [24,25,48,51]. This problem was also discussed in the studies of Decker [52] and Klich et al. [18], who showed that in areas where the European bison has not existed for hundreds of years, people base their knowledge solely on common myths about this species. Poor knowledge and fear of European bison may significantly impact human support for the restoration of this species [23,52].

The aforementioned differences in the approach to nature conservation among different social groups are extremely important considerations for practitioners of species conservation management. Nowadays, authorities (local or central) try to involve as many groups of stakeholders as possible in nature conservation management. With such a participatory approach, the issue of accurate knowledge concerning the discussions and differences in opinions becomes crucial to achieving a satisfactory consensus [24,36,42,53,54,55,56,57]. This shows that in order to balance the social approach to European bison, educational activities should be conducted not only for local residents but also for city residents to effectively inform them about the real problems experienced by village communities when coexisting with such large mammals, including the fact that conflicts may become more frequent as the numbers of European bison increase. This would allow stakeholders to gain more reliable knowledge and to more effectively facilitate discussion on planned protective measures.

It is worth noting that there was a significant number of respondents who had undecided views (i.e., "hard to say" responses) among both the city residents and the local village residents of the Bieszczady Mountains. This provides great potential for educational campaigns aimed at promoting a positive but realistic attitude to the conservation of the European bison. These types of activities should promote increased levels of up-to-date and accurate information about the impact of European bison on local communities; they should also prevent negative attitudes. In the absence of these types of appropriate educational measures, the level of social acceptance of the European bison among local residents has been shown to decrease significantly [58]. The results shown in this study described one of the first reported instances of this negative phenomenon. A significant group of local respondents (38%) were opposed to a further increase in the wisent population. This provides a clear signal to take appropriate action because, as some examples from the literature show, attitudes towards protected species can change relatively quickly from "victim to perpetrator" [59]. A lack of action may lead to a situation in which the conservation of wild populations is supported mainly by city dwellers, who do not face any direct problems resulting from coexisting with wild animals on a daily basis, and local village inhabitants’ negative attitudes will increase. The histograms will separate as a result of the greater polarization between the various social groups, as is illustrated in Figure 2 and Figure 3. Kato et al. [24] even stated that, in this context, human–human conflict is ironically an even more complicated problem than human–wildlife conflict. This is an unfavorable situation that should be avoided as it makes it very difficult to forge stakeholder agreement in management plans, as has been noted by many authors [24,36,55,56,60,61,62]. 

What is the cause of the negative attitudes towards European bison in local communities? Our study indicated that they are the result of the perceived risk of forest use limitations and the damage caused by these animals (Table 1), which becomes more important when it is happens directly to one’s own property (Figure 4). These are typical reasons that shape society’s attitude towards the presence of wild animals [25,36,41,43,62,63,64,65]. In the study by Klich et al. [18], it was suggested that if the European bison does not occur in a given area, perception of this species is likely to be shaped by other wild species; on the other hand, where the European bison is present, its management is of primary importance. Our results confirm the second hypothesis because the damage caused by the European bison, in the opinion of the local community, is quite significant compared to the frequency of damage caused by other wild species. In the Bieszczady Mountains, the European bison is now one of the main conflict-causing species, even though the usual predatory species are indicated as causing the most conflict [25,41,51,66,67,68]. Currently, the main methods of reducing European bison damage in the Bieszczady Mountains are based on feeding them in the forest during winter (which limits feeding on private farmland) and paying compensation for damage caused by them. Damage estimation and compensation payments are carried out by local government officials; however, as the results of our surveys showed, the process of compensating local residents for the damage caused by the growing bison population is not effective. Only 6% of people are satisfied with the outcomes of their claims, and most of them (62%) do not effectively report any damages at all. These results showed that in terms of compensation, there is still a lot to be done in the fight to reduce negative social attitudes towards the European bison.

The situation could be improved by conducting extensive workshops for local residents aimed at explaining how to correctly complete and submit claims. In addition to understanding the correct compensation process, it is equally important to educate people in the value of species conservation. However, it seems that these ad hoc measures are not enough to effectively minimize social conflicts in this area on a long-term basis. This is because social perception caused by forest use restrictions due to bison damage is a problem that cannot be solved by these types of administrative activities. As a previous study showed [18], even relatively high-density levels of European bison may be associated with positive acceptance of this species, but this requires active management. However, for a more long-term effect, optimal population density should be sought. A more effective solution seems to be to introduce mechanisms to further inhibit the growth of the wisent population in the Bieszczady Mountains. However, Doney et al.’s [36] study showed that when searching for an optimal management method, a conflict between the opinions of urban and rural residents may prove inevitable.

## 5. Conclusions

The Bieszczady Mountains are home to one of the largest free-living populations of European bison in the world, and the abundance of this protected species in this area is increasing. Our study showed that this increase in the European bison population is causing an increase in negative attitudes among local communities, and European bison have now become one of the main conflict-causing species in the Bieszczady Mountains (with levels comparable to wolves). Already, 38% of local residents believed that there are too many European bison in Bieszczady. People who had personally experienced material losses caused by European bison had the most negative attitudes towards this species. Material damages and access restrictions to forests were mentioned by local residents as the main threats caused by the European bison. The current system of damage compensation, which is estimated and paid by government bodies, does not solve the problem of negative attitudes towards the European bison because only 6% of people were satisfied with the compensation paid. Educational workshops can probably alleviate these conflicts in the short-term, but long-term effects can only ultimately be achieved by inhibiting further population growth. The inhabitants of Rzeszów (the largest city in the region) had significantly different attitudes towards European bison: they had a more positive attitude towards European bison conservation and wanted further population growth in the Bieszczady Mountains. These city dwellers considered that the main threat posed by bison is to public health, thereby proving that they have a very limited understanding of the real problems faced by local populations who coexist with this large herbivore. In order to develop a balanced management strategy for the growing European bison population in the Bieszczady Mountains, it is important to understand the aforementioned differences in opinions to bison between local communities and city residents. It is very important to maintain a positive social perception regarding free-living populations of European bison. Only in this way will it be possible to convince other local communities to accept and establish the new populations that will be necessary for the effective conservation of this species.

## Figures and Tables

**Table 1:** The effect of possible disadvantages caused by the bison population (health risks, economic risks, and forest use limitations); Bieszczady village inhabitants’ and Rzeszów city dwellers’ responses to questions 3, 4, and 5 on attitudes to European bison (response to question 1—Supplementary Table S1).

	Bieszczady			Rzeszów		
	B	Χ^2^	*p*	B	Χ^2^	*p*
Intercept	4.33	772.99	<0.001	4.55	566.52	<0.001
Health risks	-	-	-	−0.40	25.01	<0.001
Economic risks	−0.15	11.45	0.001	-	-	-
Forest use limitations	−0.29	39.98	<0.001	-	-	-

## Data Availability

The data presented in this study are available on request from the corresponding author.

## Supplementary Materials

The following are available online at https://www.mdpi.com/2076-2615/11/2/503/s1, Table S1: List of questions asked, answer options, and conversion to Likert scale (Likert) or to the grouping variable (Group).
