# Supplementary material for: Human–Wildlife Conflict: The Human Dimension of European Bison Conservation in the Bieszczady Mountains (Poland)
Source: Animals (Basel). 2021 Feb 15;11(2):503. doi: 10.3390/ani11020503 (PMC7919045; doi:10.3390/ani11020503)
Supplement: Supplementary file 1 [file animals-11-00503-s001.pdf]

Table S1. List of questions asked, answer options, and conversion to Likert scale (Likert) or to the grouping variable (Group).

Questions		Answers					
1.	Is the presence of the European bison in the Bieszczady Mountains beneficial for the local community?	Definitely Not	Rather Not	Hard to say	Rather Yes	Definitely Yes	
		1	2	3	4	5	Likert
2.	How many European bison should be in the Bieszczady Mountains?	Definitely Less	Rather Less	Hard to say/ as much as there is	Rather More	Definitely More	
		1	2	3	4	5	Likert
3.	Does the presence of E. bison in the Bieszczady Mountains cause any threats to human health and life?	Definitely Not	Rather Not	Hard to say	Rather Yes	Definitely Yes	
		1	2	3	4	5	Likert
4.	Does the presence of E. bison in the Bieszczady Mountains cause any risks of economic losses (cars, fences, gardens, farmlands, forests, etc.)?	Definitely Not	Rather Not	Hard to say	Rather Yes	Definitely Yes	
		1	2	3	4	5	Likert
5.	Does the presence of E. bison in the Bieszczady Mountains cause any forest-use limitations (logging trees, mushrooming, walking, etc.)?	Definitely Not	Rather Not	Hard to say	Rather Yes	Definitely Yes	
		1	2	3	4	5	Likert
4.	Did you suffer any damage from the European bison in the last 2 years?	Yes	Not	I do not remember			
		1	2				Group
5.	If so, how many times?	*Number of incidents..*					
6.	Did you claim compensation? (*number of incidents)*	Yes	Not	I do not remember	Not applicable		
		1	2				Group
7.	If so, have you received any compensation? (*number of incidents)*	Procedure interrupted	Rejected application	Too little compensation	Adequate compensation		
8.	Did you suffer any damage from other wild animals in the last 2 years?	Yes	Not	I do not remember			
		1	2				Group
9.	If so, indicate the species.	*Species…*					
10.	Did your family or friends suffer any damage from wild animals, including European bison, in the last 2 years?	Yes	Not	I do not remember			
11.	If so, indicate the species.	*Species…*					
12.	Age (years)	18–39	40–60	over 60			
		1	2	3			Group
13.	Sex	Women	Men				
		1	2				Group
14.	Are you a hunter?	Yes	No				
		1	2				Group
